# Endoscope-assisted microvascular decompression in hemifacial spasm with a teflon bridge

**DOI:** 10.1007/s00701-024-06142-7

**Published:** 2024-05-30

**Authors:** Thomas Rhomberg, Márton Eördögh, Sebastian Lehmann, Henry W.S. Schroeder

**Affiliations:** 1 Department of Neurosurgery and Neurorestoration, Klinikum Klagenfurt am Wörthersee, Klagenfurt, Austria; 2 Department of Neurosurgery, University Medicine Greifswald, Greifswald, Germany

**Keywords:** Microvascular decompression, Hemifacial spasm, Endoscope-assisted microsurgery, Retrosigmoid approach, Teflon, Operative video

## Abstract

**Background:**

Microvascular conflicts in hemifacial spasm typically occur at the facial nerve’s root exit zone. While a pure microsurgical approach offers only limited orientation, added endoscopy enhances visibility of the relevant structures without the necessity of cerebellar retraction.

**Methods:**

After a retrosigmoid craniotomy, a microsurgical decompression of the facial nerve is performed with a Teflon bridge. Endoscopic inspection prior and after decompression facilitates optimal Teflon bridge positioning.

**Conclusions:**

Endoscope-assisted microsurgery allows a clear visualization and safe manipulation on the facial nerve at its root exit zone.

**Supplementary Information:**

The online version contains supplementary material available at 10.1007/s00701-024-06142-7.

## Relevant surgical anatomy

**Fig. 1:**
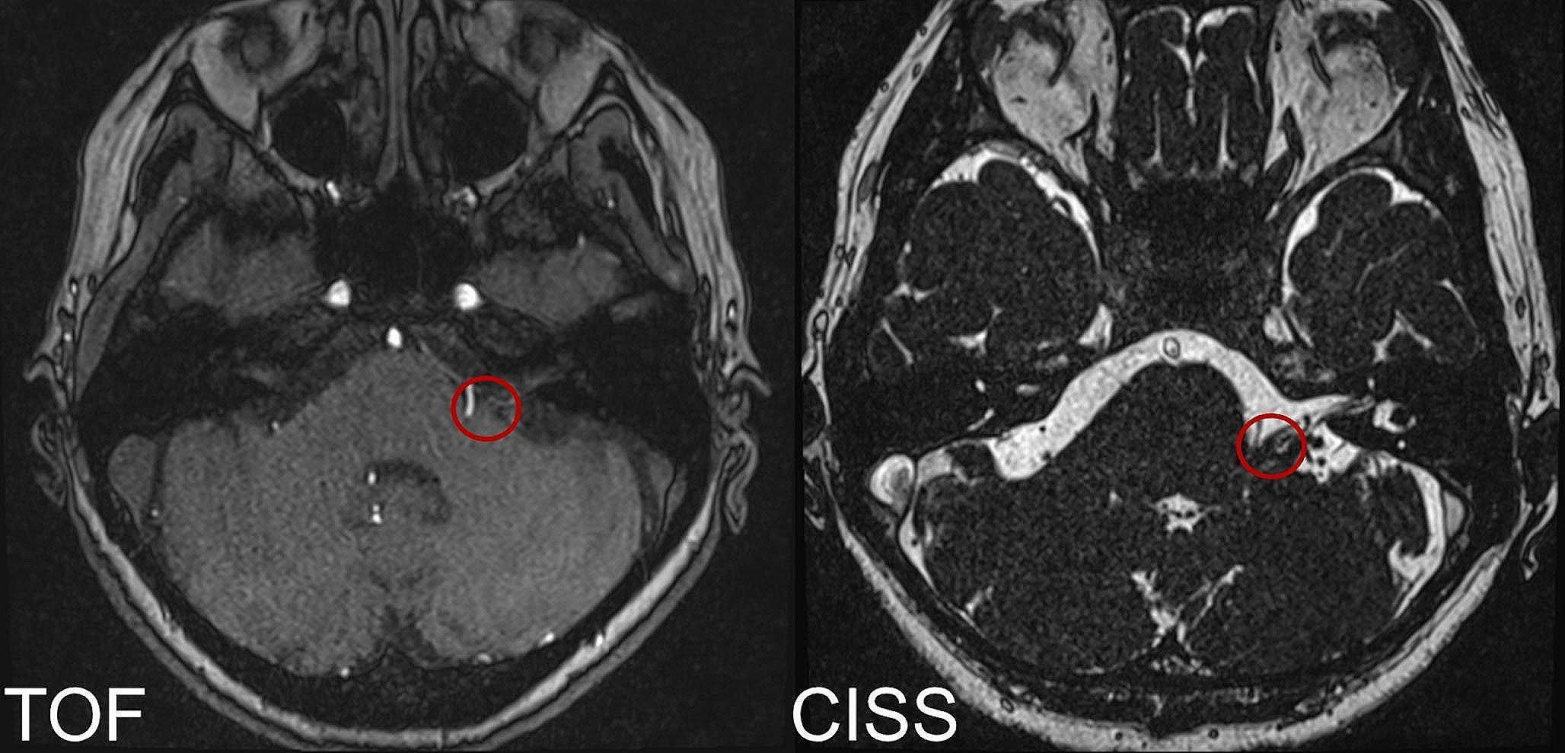
Preoperative axial TOF and CISS MRI sequences demonstrate a potential conflict (red circle) between the PICA and the left facial nerve at its root exit zone, near to the brainstem´s surface

Hemifacial spasm (HFS) is a movement disorder with involuntary contractions of the muscles innervated by the facial nerve. HFS is predominantly caused by a vascular conflict with the facial nerve [3]. In most cases, the culprit compression is located proximally at the nerve root exit zone (REZ) at the pontine surface or in the pontomedullary sulcus [2]. Accurate and detailed visualization of the nerve root exit zone is crucial in performing microvascular decompression (MVD). Most neurovascular conflicts causing HFS are related to the posterior inferior (PICA) or the anterior inferior cerebellar artery (AICA). The conflict can also be induced by the vertebral artery (VA) or a vein [8]. Multiple vascular compressions are also known. Preoperatively we assess the individual pathoanatomy using a CISS (Constructive Interference in Steady State) and TOF (Time of Flight) MRI-sequence (Fig. 1).

## Description of the technique

After general anesthesia has been induced, the procedure is performed with intraoperative neuromonitoring focusing on lateral spread responses and auditory evoked potentials. As a neuroprotective treatment, patients receive a single dose of 20 mg dexamethasone and nimodipine is administered intravenously at a rate of 1 mg/h during the first hour of surgery, followed by 2 mg/h for up to 24 h postoperatively. The upper body is elevated by 15°. The patient is positioned supine with a 45–50°-degree head rotation to the contralateral side and a sharp fixation of the head. Supportive bracings on the contralateral side enable safe tilting of the operating table for additional 30°, if necessary.

**Fig. 2:**
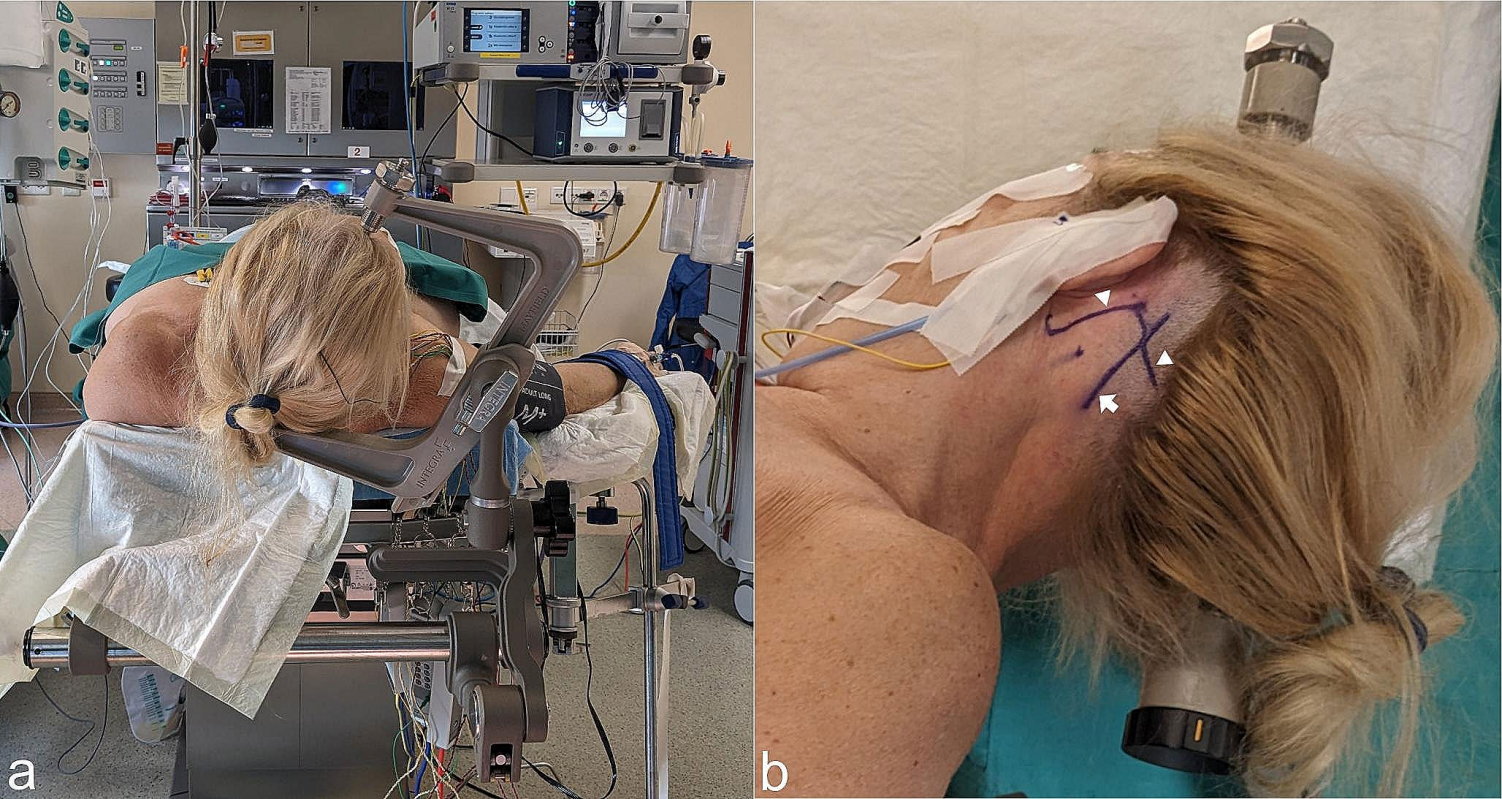
Patient positioning and surface anatomy orientation. **A**: Patient is in supine position with an elevated, slightly rotated head and sharp fixation. Intraoperatively the table is tilted up to 30° to the contralateral side. **B**: Minimal hair shaving is performed, and the course of the transverse and sigmoid sinus is outlined (arrowhead) to plan the skin incision (arrow), which is drawn as an oblique line

**Fig. 3:**
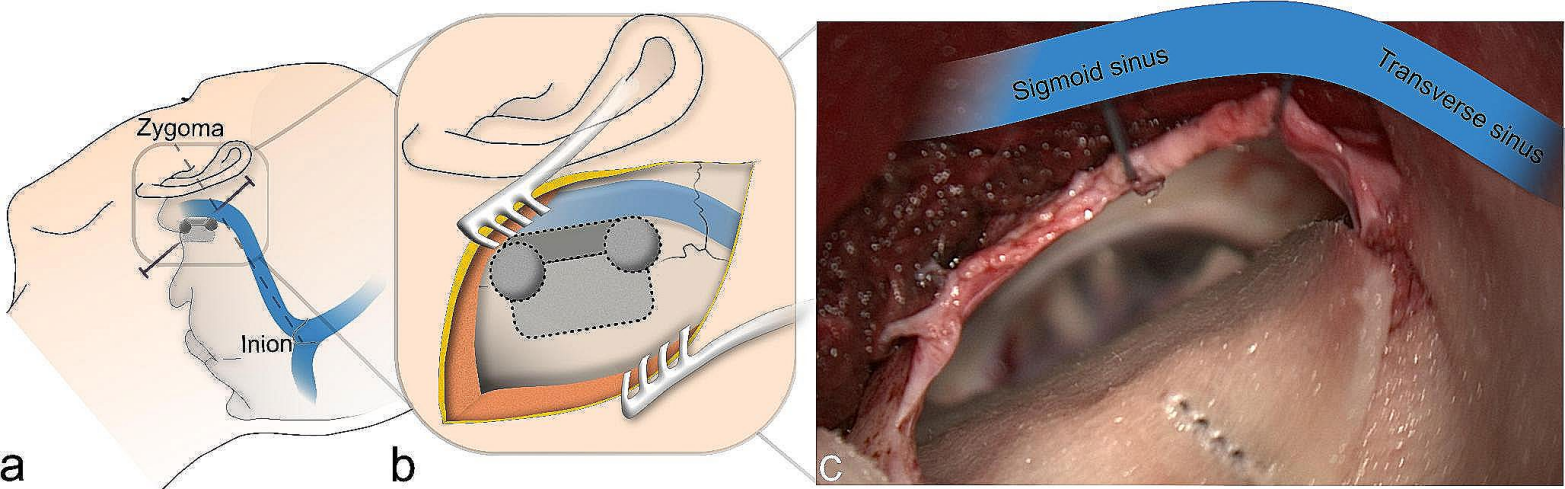
Schematic drawing of the procedure from the surgeon´s point of view, left side. **A**: Planned skin incision and craniotomy in relation to the relevant anatomical structures. **B**: Outline of the burr holes and extension of the craniotomy. The inferior burrhole may suffice. The light grey area indicates the osteoplastic craniotomy area and the dark grey area the osteoclastic extension. **C**: Intraoperative situs after dural opening. The course of the sinus projection is demonstrated

The course of the transverse sinus is located along a line drawn from the zygoma to the inion. The tip of the mastoid as well as the mastoid notch are palpated. A vertical line from the mastoid notch crosses the level of the transverse sinus where we define the junction of the transverse and sigmoid sinuses. The sigmoid sinus courses from this area to the mastoid tip. The optimal skin incision is posterior from the junction, around two fingerbreadths behind the ear (Figs. 2 and 3). The skin incision is performed as a straight oblique line of 5–6 cm length, with one-third extending cranial to the transverse sinus and two-thirds inferior to it. The muscles are dissected along a straight line that follows the trajectory of the skin incision, exposing the bone. Subsequently, a retractor is positioned to ensure optimal exposure for the craniotomy (Fig. 3). After creating one or two burr holes along the projection of the sigmoid sinus, a 2 to 3 cm craniotomy is performed. We partially unroof the sigmoid sinus with a high-speed drill and/or bone punch as an osteoclastic extension. Exposure of the transverse sinus is not necessary. The dural incision follows the sigmoid sinus course leaving 2–3 mm for later suturing. Tight dural tacking sutures are applied along the edge of the sigmoid sinus, providing an optimal surgical view into the cerebellopontine angle.


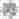


**Fig. 4:**
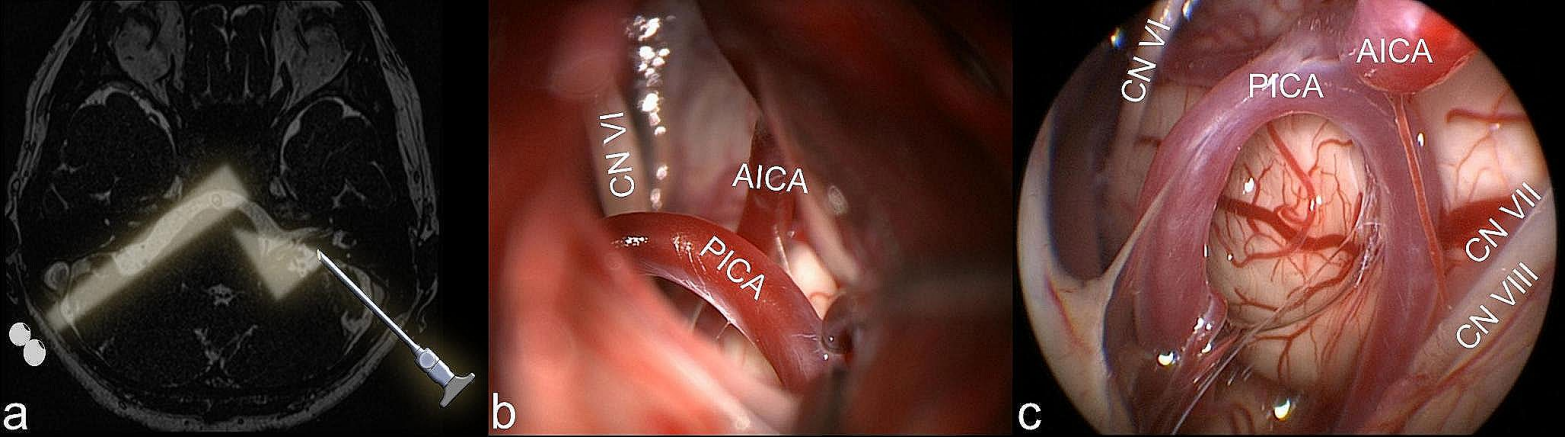
**A**: The microscopic view lacks the panoramic and angulated view of the cerebellopontine angle compared to that of a 45° endoscopic optic lens. The use of an angled endoscopic view is very helpful for proper visualization and inspection of the facial nerve’s root exit zone. **B**: View of the cerebellopontine angle with the surgical microscope. The root exit zone of the facial nerve cannot be identified. **C**: endoscopic view of the situs illustrated on “B”. 45° endoscopic optic lens offers a panoramic view into the cerebellopontine angle, allowing for direct observation of the root exit zone of the facial nerve

Due to patient positioning, sufficient cerebellar retraction can be achieved by gravity without the need of spatula. Additionally, tilting of the operating table may optimize the viewing angle. The operating microscope is initially utilized to release cerebrospinal fluid (CSF) and expose the lower cranial nerves (CN IX, X, XI), followed by CN VII and VIII, through careful subarachnoid dissection mainly using microscissors. Once a sufficient working space has been established, an endoscope with a 45° optic lens is employed to examine the pontomedullary sulcus and the root exit zone of the facial nerve to visualize the neurovascular conflict (Fig. 4) and help in choosing the individual decompression technique.

For microvascular decompression, we primarily utilize Teflon. This choice is based on our experience, which indicates that Teflon’s inert properties reduce the formation of adhesions and fibrosis compared to the use of autologous materials such as muscle. Additionally, Teflon’s versatility allows for optimal custom tailoring of pieces to precisely accommodate the unique pathoanatomy of each individual case.

Three distinct methods for Teflon placement are employed: a bridge-like structure, shredded Teflon, or a sling. The optimal approach is selected to transpose the artery away from the nerve, ensuring that no vessel is compressed and the nerve is not in direct contact with Teflon, if anatomically possible.

**Fig. 5:**
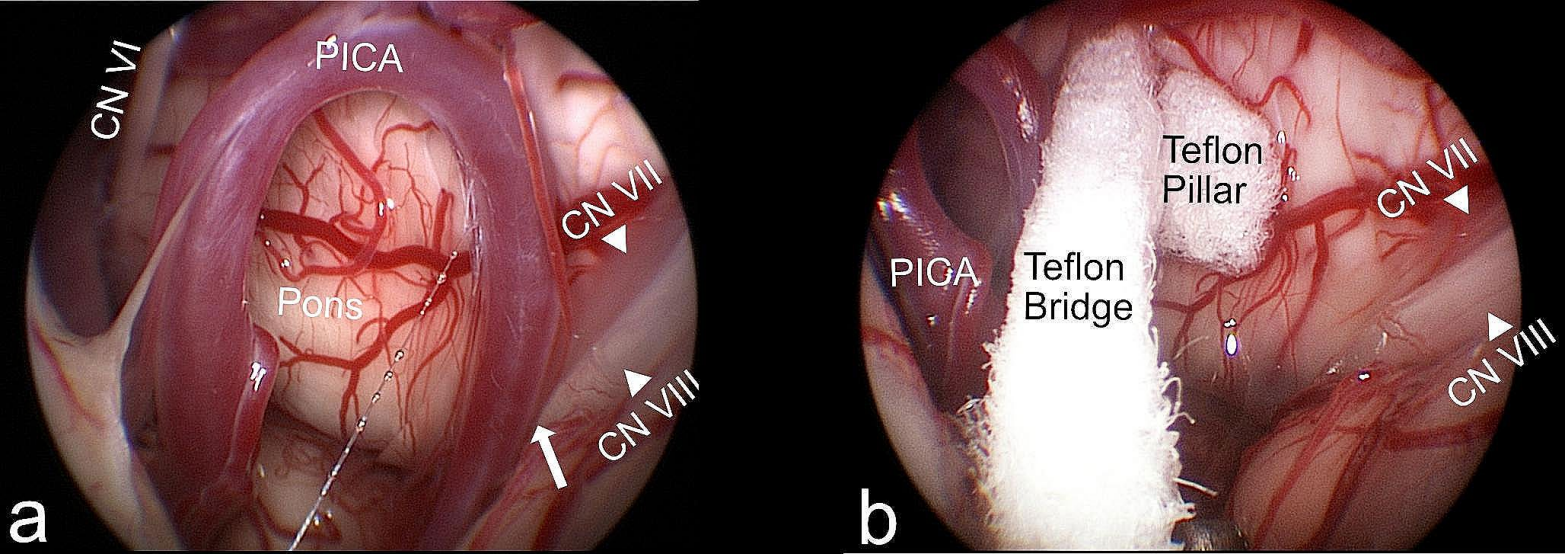
View with a 45° endoscope. **A**: A clear neurovascular conflict of the PICA with the facial nerve at the root exit zone can be observed (arrow). **B**: Image after successful microvascular decompression of the facial nerve with a Teflon bridge. Neither the vessel nor the Teflon is in contact with the facial nerve

**Fig. 6:**
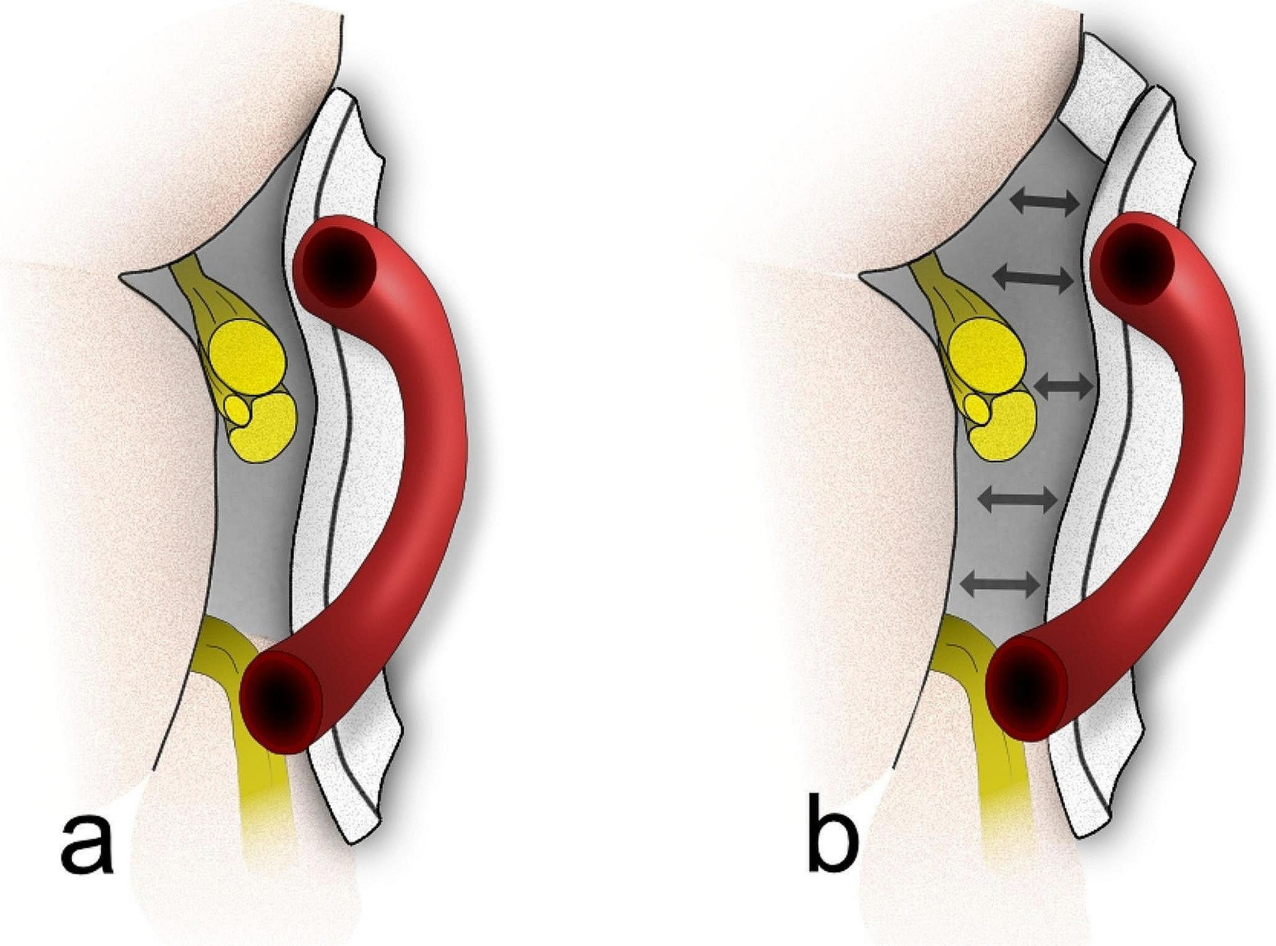
Schematic illustration of the neurovascular decompression technique employing a Teflon bridge, frontal view. **A**: depicts the use of a single piece of Teflon to create a straightforward Teflon bridge. **B**: demonstrates the same technique augmented with a pillar to further increase the distance between the Teflon and the facial nerve

In this case, a construct comprising of two Teflon pads, arranged to resemble a bridge with its pillar, has been positioned between the pons and the flocculus (Fig. 5). This technique has been described previously and was adapted to the specific requirements of our case [6, 10]. Due to the narrow operative corridor and the space required for the endoscope, the Teflon pads are positioned under microscopic sight. In certain cases, the Teflon bridge may be placed without a pillar when there is enough space between the Teflon bridge and the facial nerve (Fig. 6). After meticulous inspection of the neurovascular decompression, fibrin glue can be applied to the Teflon construct to provide additional stability.

### Indications

The discussion on the indications for MVD in HFS is beyond the scope of this article. Nonetheless, in all cases of HFS, we employ an endoscope-assisted microsurgical technique to ensure proper visualization of the root exit zone of the facial nerve.

### Limitations

Only patients with a relatively flexible cervical spine are suitable for a supine position as the head rotation is necessary for a sufficient overview of the surgical situs.

### How to avoid complications

The mortality rate associated with the procedure is very low, at approximately 0.1% [9]. However, other related complications can profoundly affect the patient’s quality of life:

- CSF leakage should be avoided at all costs. Thus, meticulous watertight dural closure, along with sealing any open mastoid cells using muscle tissue, and fibrin glue is essential.
- Adhesions between Teflon and the facial nerve can, in itself, induce HFS [1], although we have never seen granuloma formation. Therefore, to reduce the potential risk of needing surgical re-exploration due to recurrence of HFS, it is recommended to avoid such contact whenever possible.
- The head should be rotated to the maximum extent possible without compressing the contralateral jugular veins.
- Retraction of the cerebellum should be minimized to prevent stretching of the vestibulocochlear nerve, thereby reducing the risk of postoperative hearing loss.

## Specific information for the patient

Patients should be made aware of potential specific postoperative deficits. These may include temporary hoarseness resulting from irritation of the lower cranial nerves. Additionally, although infrequent, complications such as facial palsy and hearing loss may occur if the facial or vestibulocochlear nerves are irritated. Around 14% of the patients develop a delayed postoperative facial palsy, which is typically transient and resolves within weeks following the surgery [4].

**10 key point summary**.

1. Position the patient supine with the head rotated 45–50° contralaterally and secured in a Mayfield clamp. Utilize supportive bracing on the side to facilitate safe tilting of the operating Table.
2. Setup includes neuromonitoring with auditory evoked potentials and lateral spread response monitoring.
3. An oblique skin incision is used to perform a small retrosigmoid craniotomy. The dura is opened parallel to the sigmoid sinus.
4. Subarachnoid dissection is performed with the operating microscope.
5. Inspection with a 45° endoscope to visualize the neurovascular conflict.
6. Depending on the appearance of the neurovascular conflict, a Teflon construct resembling a bridge, sling or a piece of shredded Teflon is used for MVD [5, 7].
7. Verify the absence of a lateral spread response if one was initially observed at the surgery’s onset.
8. Application of fibrin glue to provide additional stability to the Teflon bridge.
9. Watertight dural closure and ensure meticulous sealing of any opened mastoid cells.
10. Intermediate care unit for one night observation and postoperative CT scan on the following day. It is aimed to discharge the patient on day 4 to 5 after surgery.

## Electronic supplementary material

Below is the link to the electronic supplementary material.

Supplementary Material 1

## Data Availability

The original video material used for this study are available from the corresponding author upon reasonable request.
